# Characteristics of transient charge on Datong coal sample surfaces with different cracking propagation

**DOI:** 10.1371/journal.pone.0229824

**Published:** 2020-03-09

**Authors:** Jing Li, Cheng Guan, Ke Han, Zhen Wang

**Affiliations:** College of Emergency Management and Safety Engineering, China University of Mining & Technology, Beijing, China; Massachusetts Institute of Technology, UNITED STATES

## Abstract

Using an analysis of the uniaxial compression process of Datong coal samples, the change of transient charge signals on coal surfaces is observed, and the influence of sampling directions (perpendicular to bedding planes and parallel to bedding planes) on the transient charge signals is studied. The intensity in perpendicular to bedding planes is 4.6~10.2 MPa, parallel to bedding planes is 2.1~5.3 MPa. The results show that the change of the charge signals on sample surfaces is instantaneous and pulsing, and such a change is always in accord with stress change and the alternation of positive and negative charge occurring over a short time period. Under uniaxial compression, the surface charge signal characteristics of coal sample in perpendicular to and parallel to the bedding are different. With a higher value of limiting stress, the transient charge signals on coal sample surfaces perpendicular to the bedding exhibit higher strength than those of coal samples oriented parallel to the bedding. However, the number of signal pulses during the failure process, for the samples perpendicular to the bedding, is less than that for the samples oriented parallel to the bedding. According to the variation law for transient charge signals on coal surfaces, we conclude that changes in the transient charge can serve as a tool to characterize crack propagation within coal specimens and provide an important reference for the prediction of coal and rock dynamic disasters.

## Introduction

Coal and rock dynamic disasters are a rapid dynamic process of coal and rock failure under the combined action of crustal and mining stress [1]. In the deformation process, the internal energy in coal and rock is released in a variety of forms, including elastic energy, sound, heat, electromagnetic and other forms [2–4]. Based on the precursor signals from the energy release process, which are related to the stress state and loading history, a series of geophysical methods to detect coal and rock dynamic disasters, such as microseisms, acoustic emissions, and infrared and electromagnetic radiation, has been developed. For electromagnetic radiation (EMR), numerous laboratory and field studies of deformation and rupture in coal and rock have been conducted in recent decades [5–9]. The mechanism of electromagnetic radiation indicates that free charge is generated during the deformation process of coal and rock and that the movement of a free charge will lead to electromagnetic radiation. The experimental results show that there is a more basic and direct relationship between the free charge signal and coal and rock deformation [10–11]. Therefore, the study of free charge generation and variation can not only be used to analyse the mechanism of electromagnetic radiation at the microscale level, but also provides a new method for predicting coal and rock dynamic disasters.

The phenomenon of charge generation during the deformation process of coal and rock masses has been described in numerous published studies, most of which focus on the following three aspects: (1) Surface potential in coal and rock under uniaxial compression, tensile and three-point bending load has been detected via a conductive electrode [12–15] and exhibits a good correlation with changes in stress and strain, as well as surface microcurrents [16–18]. (2) The induced charge generated during the deformation process can be measured using a non-contact charge sensor, and the characteristics of charge signals in granite, sandstone and coal are discussed in the literature [19–22]. Based on the electromagnetic emission observed during the failure process, a compressed atomic model is proposed [23–24]. (3) From theoretical research, the electromagnetic field theory and double layer theory are used to calculate the amount of charge generated when coal and rock samples fail [25–27].

Most earlier studies addressing the experimental results indicate that charge signals and surface potential are generated during the destruction process of coal and rock by considering them to be isotropic materials; this, however, is not the case. Coal is anisotropic, and there are obvious structural differences in the directions that are oriented perpendicular to and parallel to the bedding planes because of tectonic action [28–30]. Few published information is available on the generation and change of free charge in coal deformation process, i.e., the transient charge signals, on the deformed surface of coal with different cracking propagation during uniaxial compression. The physical and theoretical mechanisms leading to the experimental results need further study. In this paper, a series of uniaxial loading experiments of raw coal samples, with perpendicular and parallel cracking propagations, were carried out. The current study is undertaken not only to improve the current understanding of the generation of transient charge signals but also to discuss the influence of the cracking propagation in coal on free charges.

## Experiment

### Experimental setup and sample preparation

**Fig 1:**
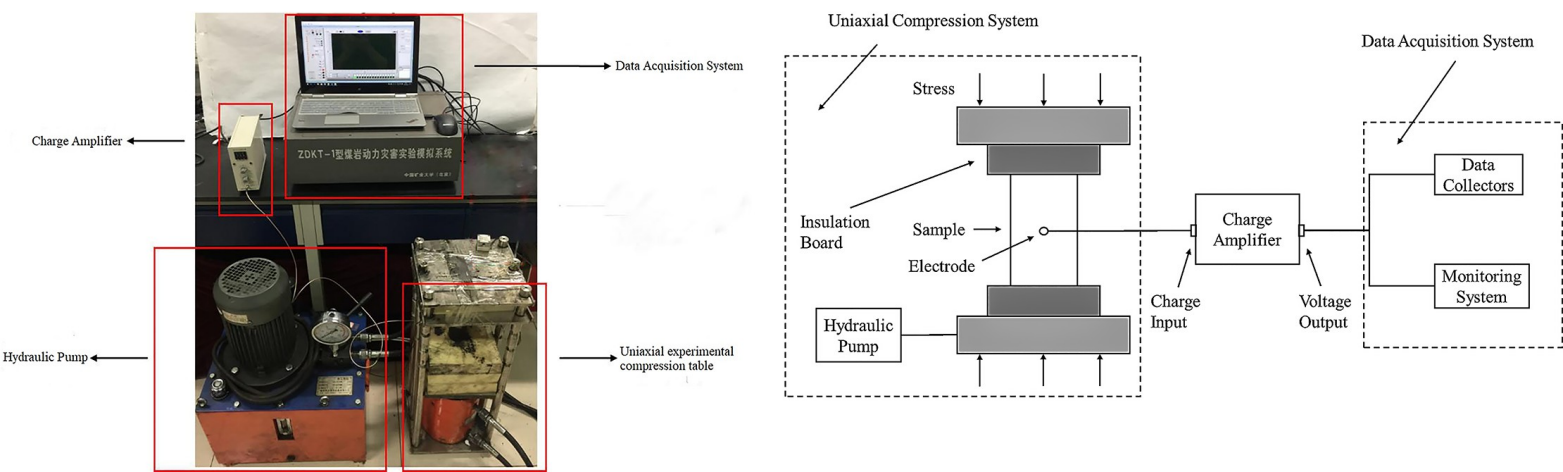
Schematic of the uniaxial stress-induced surface charge experimental setup.

The experimental setup consists of a press machine, insulating boards, charge amplifier and data acquisition system. The schematic diagram of experimental device are shown in Fig 1, the transient charge signals generated on the surface of coal samples under uniaxial compression can be quantitatively measured and analyzed. The OBS2 type hydraulic pump with a digital pressure gauge is used as the press machine compresses the sample. This pump can be used automatically or manually, the rated pressure of high pressure is 80 MPa.The digital pressure gauge is used to monitor the stress throughout the experimental process, the measuring accuracy can reach 0.05%. Two insulating boards are placed at the contact surfaces between samples and the homemade uniaxial compression test bed to ensure that the charge signals were not affected by the setup.

As the charge signals generated during the deformation process of the coal sample are relatively weak, it is necessary to use a charge amplifier with high sensitivity and amplification to collect and amplify the charge signals. In this experiment, the SD1431 charge amplifier, frequency range from 0.3 Hz to 100 KHz, produced by the Beidaihe Practical Electronic Technology Research Institute is used. The correspondence between the input charge signal and the output voltage signal of the charge amplifier is: 1 pc·mV^-1^, meaning that when the amount of generated charge signals on the surface of sample changes by 1 pc, the corresponding change in output voltage is 1 mV.

The data acquisition system is the ZDKT-1 system, developed by China University of Mining and Technology (Beijing), which contains NI 9219 and NI 9234 data collectors from National Instruments Co., Ltd. The NI 9219 collector can be used to measure stress and strain, while the NI 9234 collector can be used to analyse the frequency and amplitude of acoustic emissions along with electromagnetic and charge signals.

Test blocks of coal were obtained from the No. 3 Coal Seam in the Jurassic Coalfield from the Shanxi Datong Coal Industry Group. The Datong coals have been characterized comprehensively and have been used as standard and reference samples in numerous studies [31–32]. Table 1 lists the corresponding petrographic information for the coals used for uniaxial compression in this study.

**Table 1:** Datong coal samples: Properties used for uniaxial compression experiments.

VR_r_ (%)	Rank	Liptinite (%)	Vitrinite (%)	Inertinite (%)	Ash (%)	H_2_O (%)	V_daf_ (%)
0.64	mvb	0.8	74.6	24.6	7.4	4.15	31.0

Volatile matter and ash are calculated on a dry basis. Data adapted from [31].

**Fig 2:**
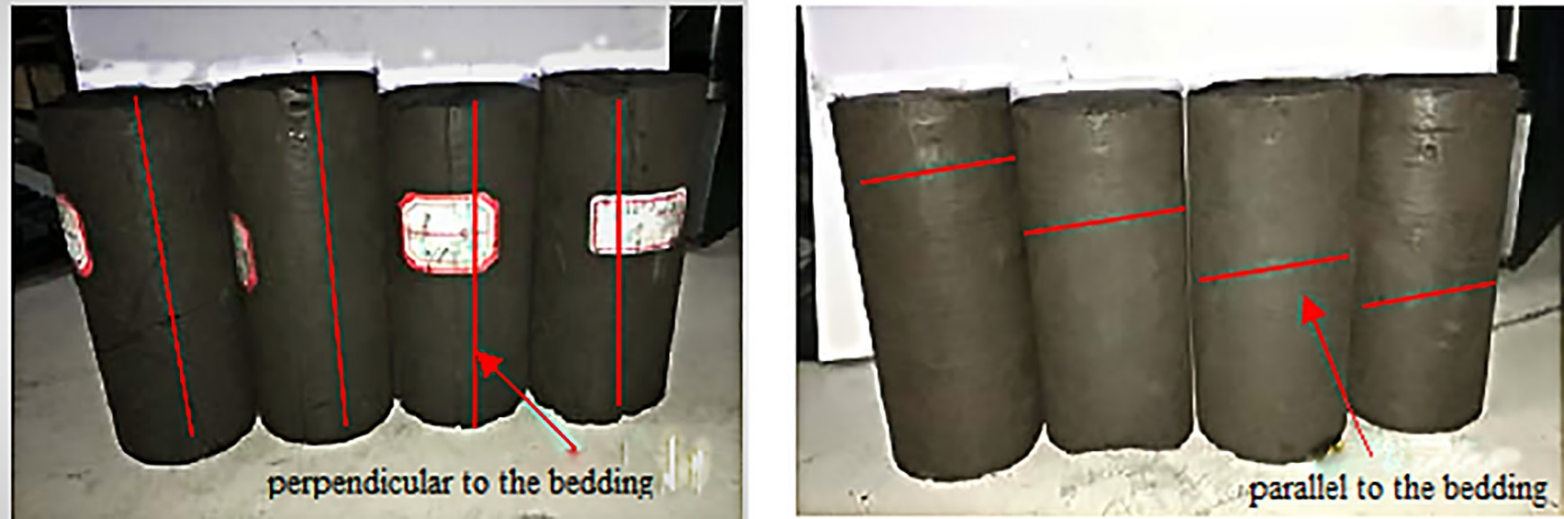
Samples with perpendicular and parallel cracking propagation.

**Fig 3:**
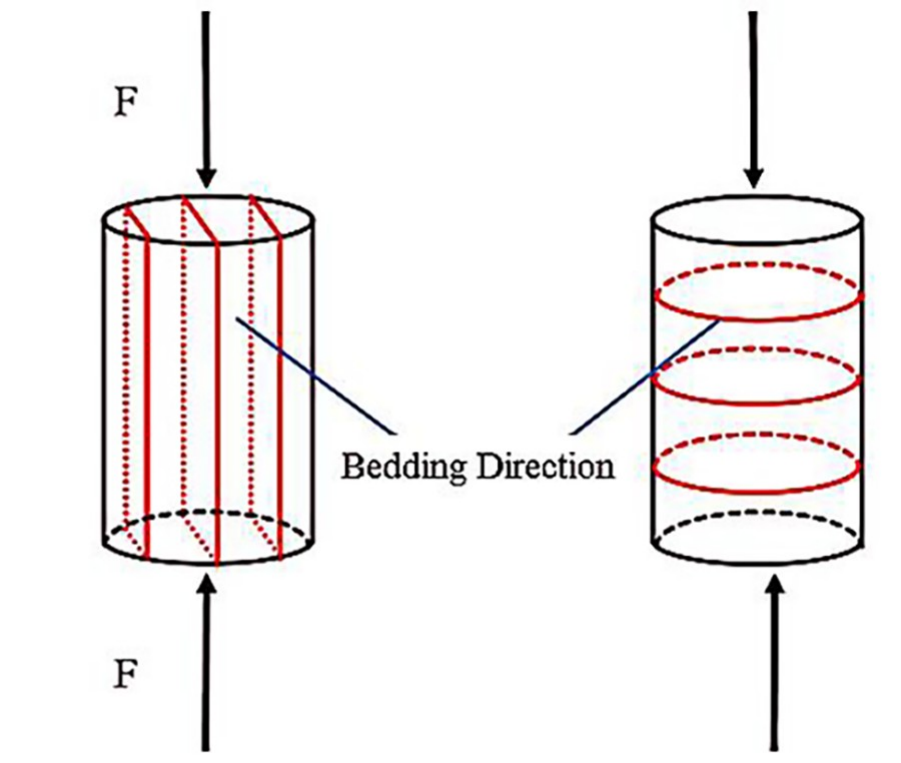
Bedding distribution in coal samples.

To analyse the effect of different cracking propagation on the transient charge signals on coal surfaces in the deformation process, the raw coal samples used in this experiment were divided into two groups: samples oriented perpendicular to the bedding and samples oriented parallel to the bedding. To demonstrate repeatability of this experiment, four samples for each group were prepared for repeated tests. As shown in Fig 2, the four samples in Fig 2(A) are the samples oriented perpendicular to the bedding and the four samples in Fig 2(B) are the samples oriented parallel to the bedding. Fig 3 shows the bedding distribution in coal samples in different groups. All the coal samples are made as Φ50×100 mm cylindrical standard specimens and marked with labels, detailed information on coal samples is shown in Table 2. Prior to the experiment, the cylindrical samples are preserved in an environmental chamber.

**Table 2:** Detailed parameters of the raw coal samples.

Sample No.	cracking propagation	Sample Size	
		Diameter (*mm*)	Height (*mm*)
**v-1**	perpendicular	50.10	99.70
**v-2**	perpendicular	50.40	99.30
**v-3**	perpendicular	50.20	99.70
**v-4**	perpendicular	50.60	100.50
**p-1**	parallel	50.40	98.50
**p-2**	parallel	51.50	99.60
**p-3**	parallel	50.00	100.10
**p-4**	parallel	50.60	99.80

### Experimental procedure

The experimental steps were as follows:

1. The coal sample was secured in the compression test bed, and two insulating boards were placed on the contact surfaces between samples and the test bed to ensure that the charge signals were not affected by the experimental setup. A copper electrode was attached in the central position of the side surface using conductive adhesive to connect the sample to the charge amplifier.
2. To eliminate the influence of static electricity caused by the sample, the surface of the sample was covered with a copper mesh and connected to ground. The copper mesh remained in place until the data acquisition system was turned on.
3. Before stress loading, environmental noise was collected and stored for 30 s and was used to offset the noise signal contained in the measured charge data collected during the deformation process.
4. The hydraulic pump was turned on in manual mode, and the data acquisition system was programmed to take readings at a frequency of 1652 Hz until the sample completely ruptured.
5. After each sample ruptured, the status of all instruments was checked to ensure their accuracy. Steps 1–4 were then repeated on all samples in the two groups.

## Results

### Different destruction forms of coal samples with different cracking propagation

**Fig 4:**
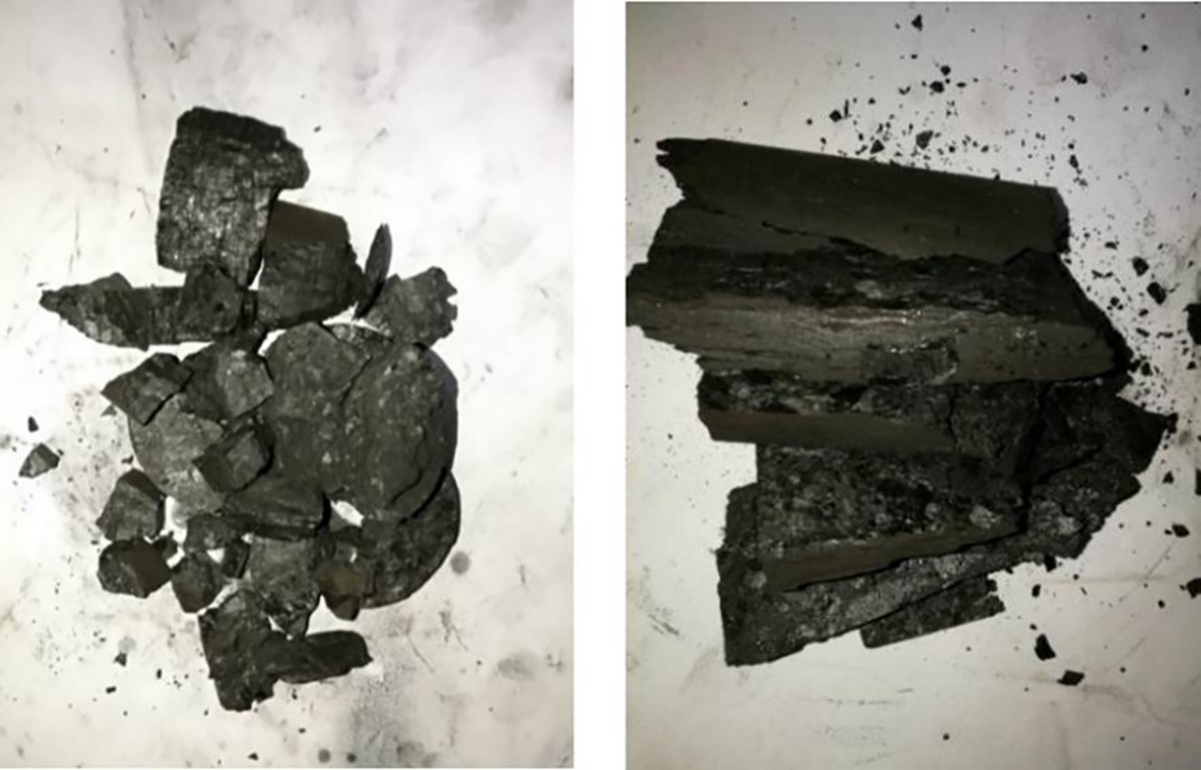
Damaged samples under uniaxial loading.

As shown in Fig 4, the destruction forms of the coal samples having different cracking propagation are completely different under uniaxial compression. When the bedding direction is parallel to the loading direction, coal sample exhibit the characteristics of splitting failure, namely, sliding-shear failure along the bedding. This is because the weak cementation between the bedding planes of coal sample, which is equivalent to the internal structure weak plane, shear strength between bedding planes is much smaller than that of other non-bedding planes. With the increase of loading time, shear force between the bedding planes gradually increases, when it is greater than shear strength of the bedding plane, coal sample will have sliding shear failure along the bedding plane. When the bedding direction is perpendicular to the loading direction, no obvious crack propagation phenomenon appeares during the loading process, when stress reaches the compressive strength of coal sample, coal sample exhibit brittle fracture of high fragmentation and small blockiness.

In Fig 4(A), the samples perpendicular to the bedding completely rupture during the compression process. The standard cylindrical specimens quickly burst into small lumps, accompanied by a loud noise as the uniaxial stress reached its peak value and the small coal lumps are ejected from the compression test bed in random directions. Fig 4(B) shows the deformation results of the samples oriented parallel to the bedding under uniaxial stress. Unlike the samples oriented perpendicular to the bedding, these samples show a gentler destruction form, in which a splitting rupture along the bedding direction takes place. The coal sample is less damaged and remains relatively intact without ejection of small lumps and without loud noises being generated during the deformation process.

### Comparison of the transient charge signals in different coal samples

**Fig 5:**
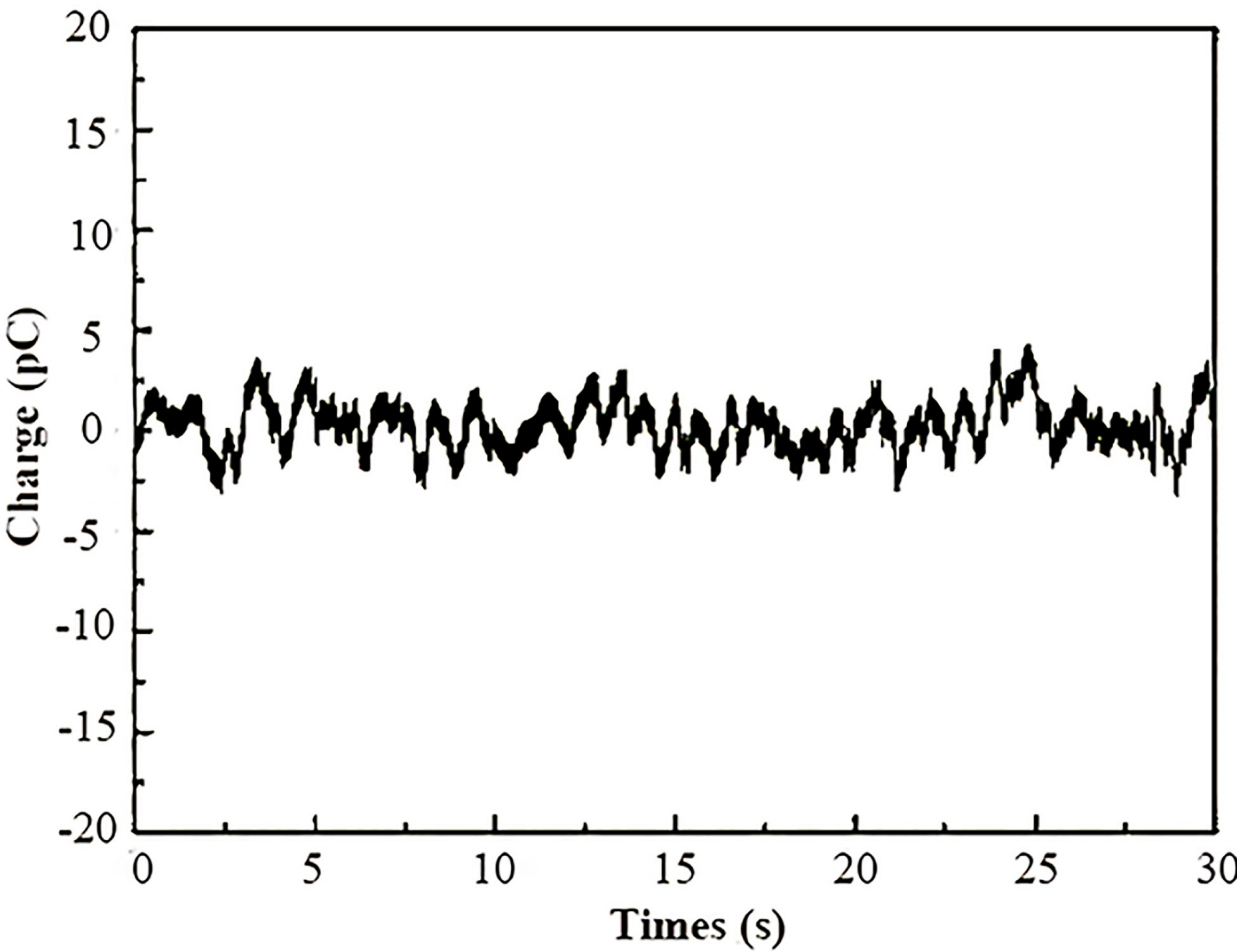
Environmental noise signals.

Prior to each uniaxial compression tests, the data acquisition system was turned on to collect 30 s of environmental noise signals, as shown in Fig 5. The noise signals came from the surrounding environment or from instrumental effects and exhibited a certain variation trend. It is clear that the intensity of the noise signals received by the charge amplifier is less than 5 pc, which has little influence on the charge signals measured during the deformation process. For the next step of data analysis, the environmental noise signals are removed first from the original charge signals, followed by an analysis and discussion of the effective charge signals.

**Fig 6:**
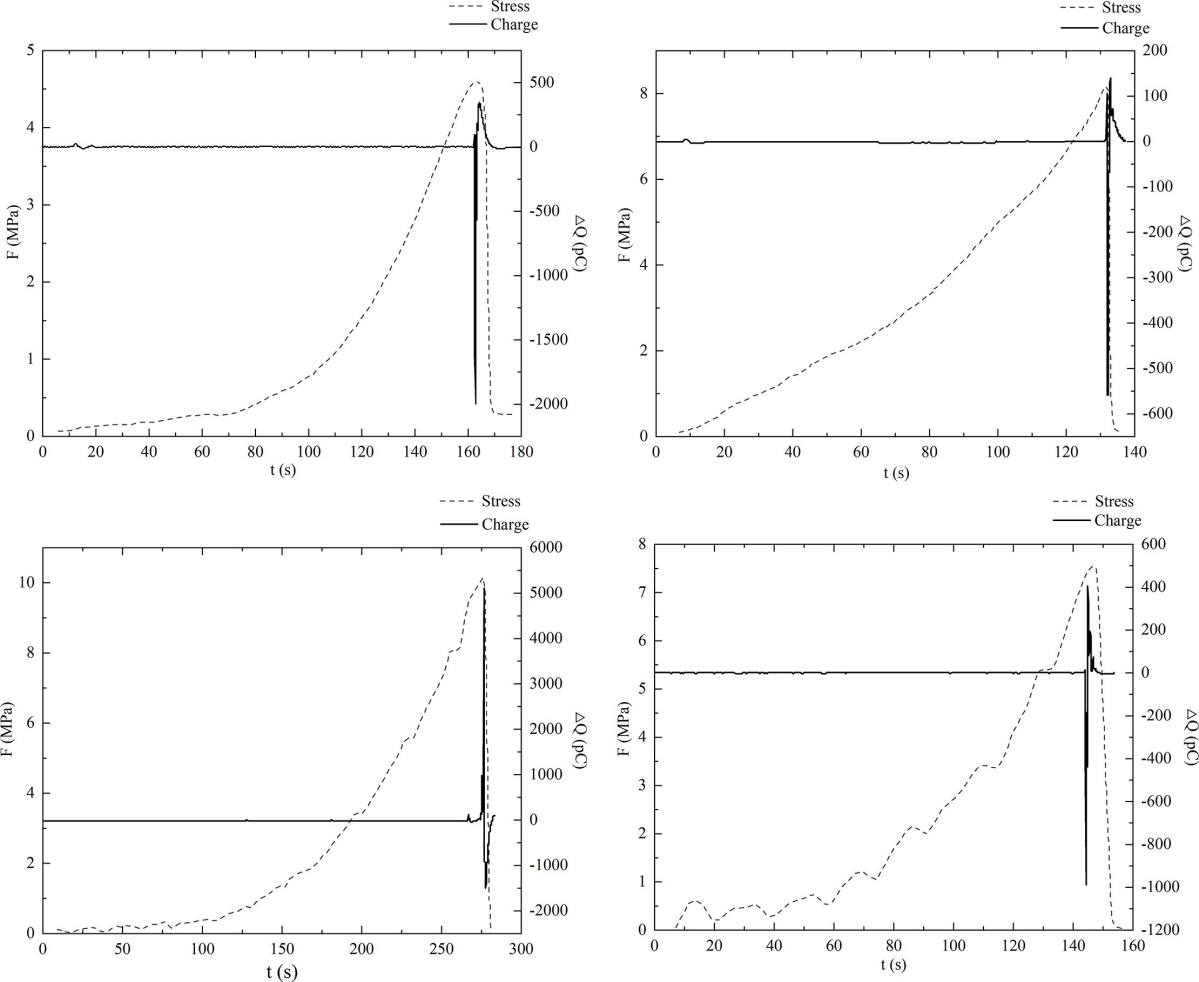
Charge signals and loading stress vs time for the samples oriented perpendicular to bedding.

**Fig 7:**
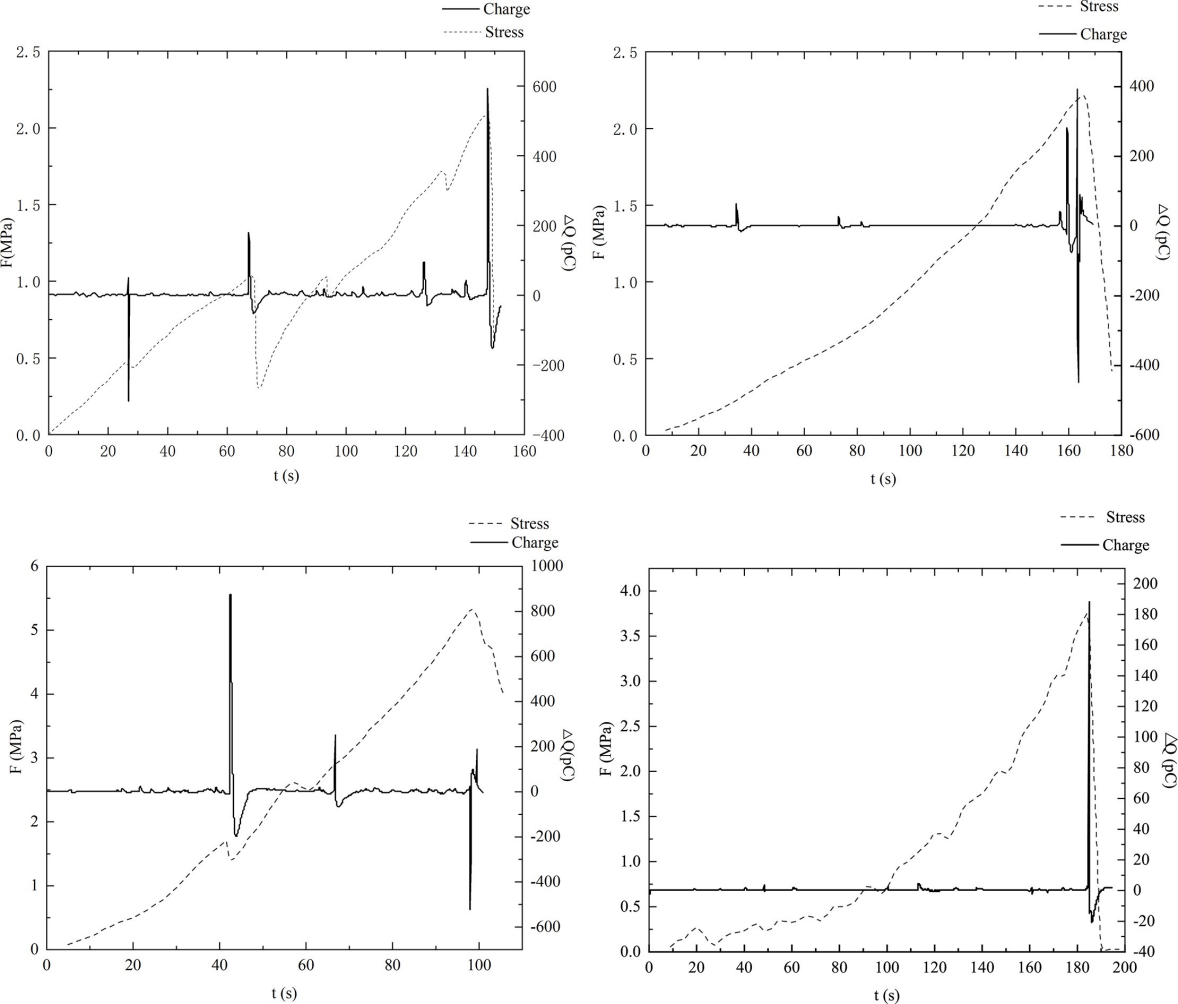
Charge signals and loading stress vs time for the samples oriented parallel to bedding.

In this experiment, eight sets of transient charge signals on the coal surfaces were collected in which the environmental noise signals were excluded. Fig 6 and Fig 7 illustrate the charge signals (*ΔQ*) and the loading stress (*F*) as functions of time for samples oriented perpendicular to and parallel to the bedding, respectively.

From Figs 6 and 7, we observe that transient charge signals are generated on the surfaces of both two groups of different coal samples during the deformation process. The charge signals are transmitted through the copper electrode at the centre of the sample and are acquired by the charge amplifier and the data acquisition system.

**Fig 8:**
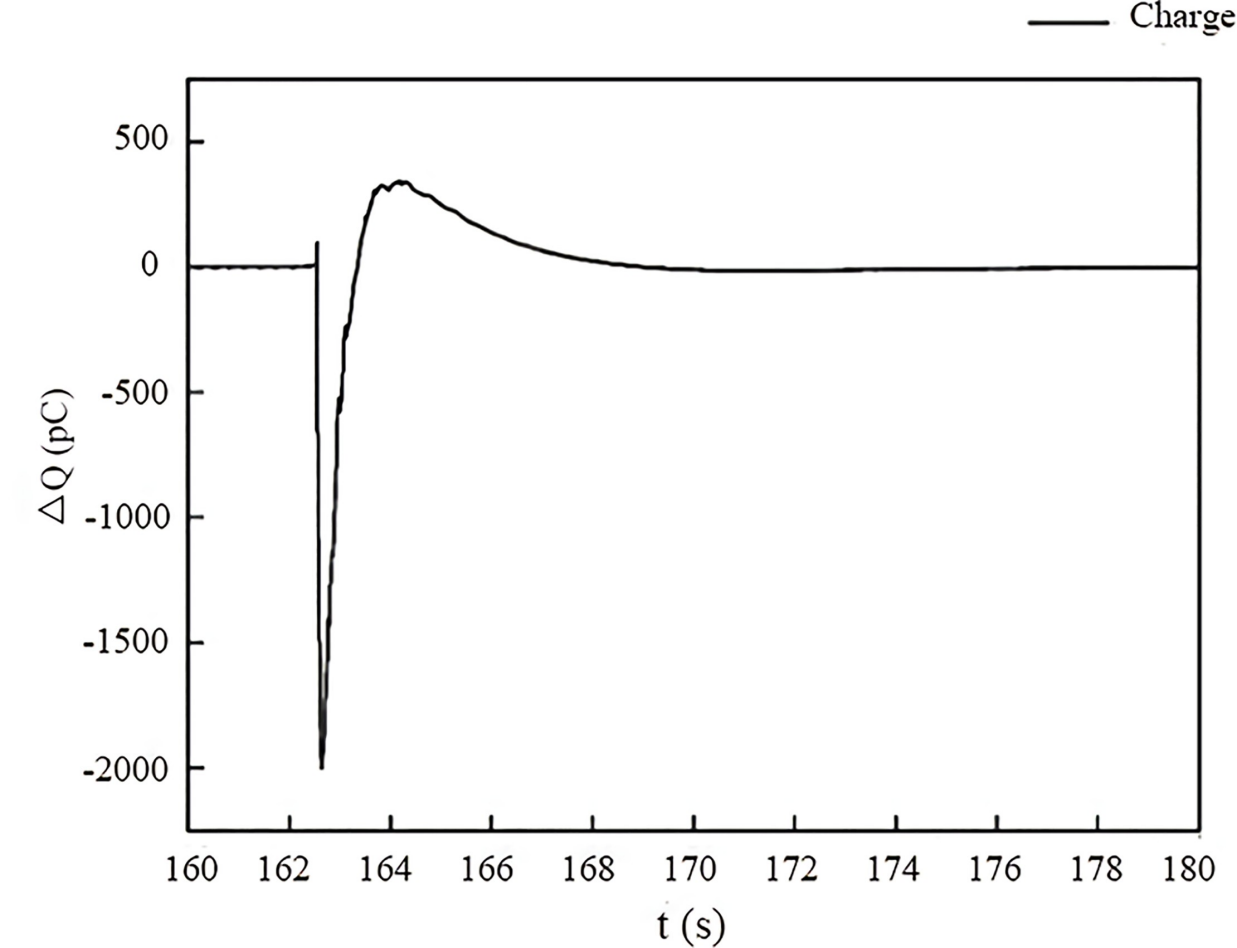
One single pulsing charge signal in sample v-1.

It is evident that the generated charge signals are instantaneous and pulse during the compression process. When the loading stress reaches its peak value, the amplitude of the charge signals also reaches a maximum, followed by an alternation of positive and negative charges over a short period of time. After the coal samples break into pieces, the amplitude of the charge signal decreases at a relatively low rate and decreases to zero over time. Comparing Fig 6 and Fig 7, it can be seen that the charge signals are quite different in the samples with different cracking propagation in the loading process. However, the single pulsing signal generated in an extremely short time shows a similar trend. Fig 8 shows one transient charge signal generated in sample v-1 when the sample is ruptured completely. The amplitude of the charge signal reaches a maximum of -2000 pc instantly, followed by a rapid decrease to zero. Then, the polarity of the charge changes to positive, indicating that there are positive charge signals generated on the coal surface. However, the amplitude of the positive charge is much smaller than that of the negative charge, and the charge signals finally dissipate to an equilibrium state, as there is no more charge generation. In this paper, the complete changing process of the signal as discussed above is defined as an effective pulse, where only the charge signals with an amplitude of larger than 5 pc are regarded as valid signals for the data analysis; those signals smaller than 5 pc are ignored.

Figs 6 and 7 illustrate the transient charge signals generated in the samples oriented perpendicular and parallel to the bedding, respectively. In this subsection, we compare the differences in charge signals between the two types of coal samples and discuss the changes in corresponding stress during the entire loading process.

Fig 6 shows that the number of effective charge signal pulses in the samples perpendicular to the bedding is small and that the corresponding stress increases smoothly and without any abrupt change at the beginning of the compression process. When the main rupture occurs in the coal samples, both the charge signals and stress reach peak values and change dramatically over time. Using the v-1 sample as an example, the loading stress increases approximately linearly to 0.28 MPa from 0 s to 60 s without obvious signal generation, except for two minor changes from 10 s to 20 s. From the recorded test video, we see that a few small cracks are formed on the surface near the copper electrode from 10 s to 20 s and then close under the uniaxial stress without further propagation. From 60 s to 163 s, the stress builds rapidly to its peak value, and only a few weak charge signals are generated. There are no corresponding cracks or other damage observed during this stage. The loading stress reaches a peak value of 4.6 MPa at 163 s followed by a sudden drop from 165 sto 170 s, indicating the sudden failure of the sample. The transient charge signals are generated at 162.5 s and reach a maximum of -2000 pc within 0.1 s, then decrease to zero, indicating that a large negative charge has accumulated around the copper electrode during this period. From 163.4 s to 170 s, the polarity of the charge signals on the surface changes to positive, and the amplitude of the signals increases to 343 pc at 164 s and then decreases to zero. This means that a certain level of positive charge is generated around the electrode, but the amplitude is much lower than the previously generated negative charge. The transient charge signals generated in the other three samples perpendicular to the bedding are similar to that in sample v-1 with respect to overall trend, but there are some differences in the signal amplitude and polarity change between the positive and negative charges, which are due to the differing properties of the coal samples.

Compared to the charge signals in the samples perpendicular to the bedding, there is a significant difference in the surface charge signals for the samples parallel to the bedding. As shown in Fig 7, there are more obvious and intense signals during the entire loading process, especially during the initial loading period. These charge signals generated before the main rupture show higher amplitudes and a better correlation with loading stress than those of the samples perpendicular to the bedding. Using sample p-1 as an example, stress increases linearly in the initial period of 0 s to 69 s, and the corresponding surface charge signals fluctuate in the range of -5 pc to 10 pc. Two slight drops in the stress occur at 27 s and 67 s, indicating that the sample has broken at those times, and the corresponding charge signals are -300 pc and 170 pc, respectively. From 69 s to 147.5 s, the stress continues to increase linearly, but the growth rate is larger than that of the previous loading stage. With an increase in stress, some small ruptures occur in the sample, along with simultaneous fluctuations in surface charge signals. At 147.5 s, the stress peak is reached, followed by a sudden decrease after 0.5 s. During this period, the surface charge signals still show small fluctuations, but their amplitudes are within -10 pc to 30 pc, which are larger than the fluctuation range in the previous stage. There is also good correlation between the charge signals and the two small reductions in stress at 93 s and 132 s. The charge signals suddenly increase to a peak of 590 pc at 147 s and then sharply decrease to a negative charge of -153 pc, followed by a gradual return to equilibrium. The variations of the surface charge signals generated in the other three samples oriented parallel to the bedding exhibit a similar overall trend.

### Influence of cracking propagation on charge signal parameters

**Fig 9:**
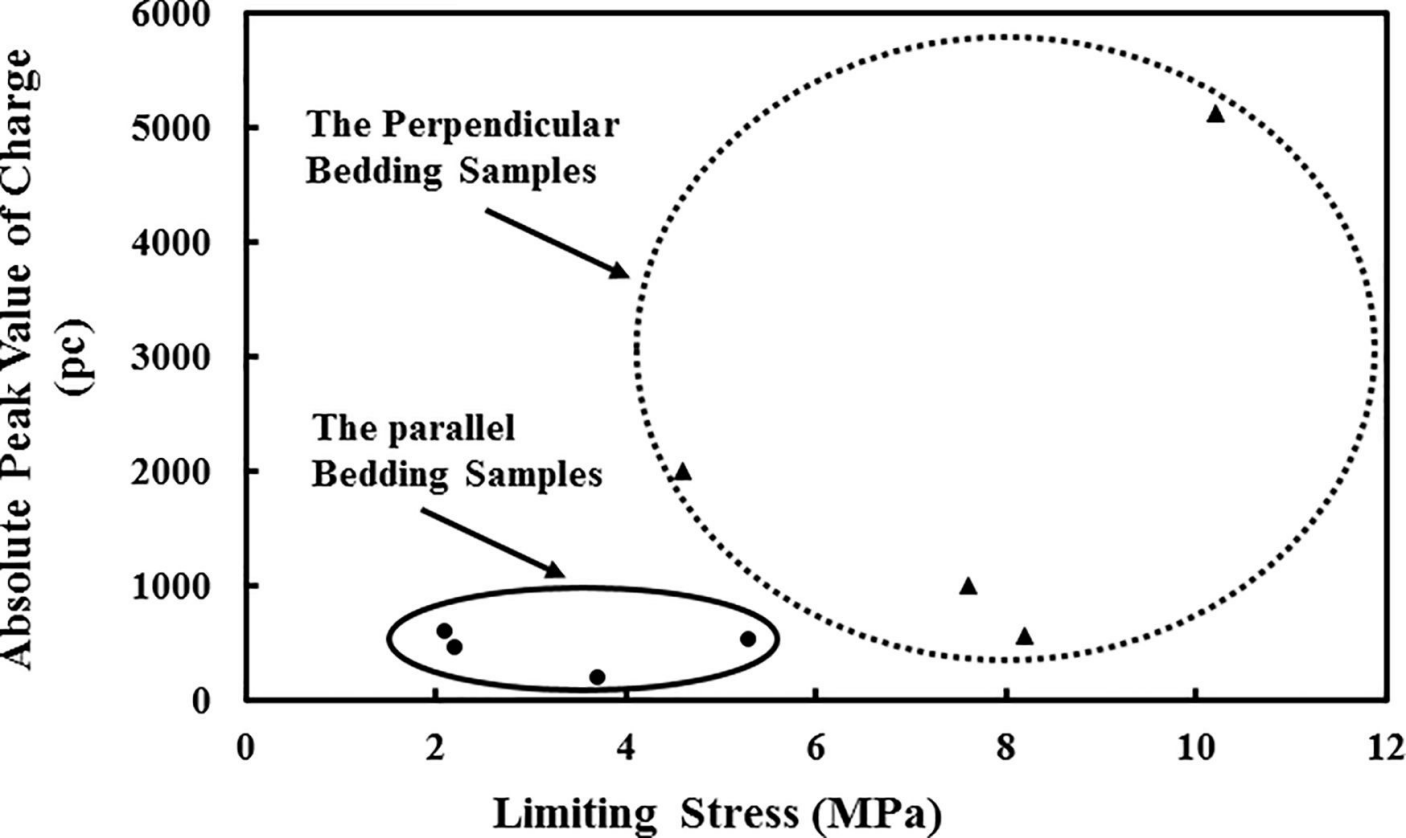
The relationship between the absolute value of the charge signals and limiting stress.

From the observations discussed above, it is clear that the generation, accumulation and decay of the surface charge signals are different due to the different cracking propagation. To clearly analyse the influence of bedding direction on the characteristics of surface charge signals and related parameters, including the limiting stress, peak value of the surface charge signal, and number of effective pulses, we list the values of these parameters in Table 3 and plot the absolute value of the charge signals versus the limiting stress for each coal sample as shown in Fig 9.

**Table 3:** Parameters for different samples.

No.	Bedding Direction	Limiting Stress (*MPa*)	Peak Value of Charge Signal (*pc*)	Number of Effective Pulses
**v-1**	perpendicular	4.6	-2000	3
**v-2**	perpendicular	8.2	-560	4
**v-3**	perpendicular	10.2	5117	3
**v-4**	perpendicular	7.6	-994	2
**p-1**	parallel	2.1	590	32
**p-2**	parallel	2.2	-450	15
**p-3**	parallel	5.3	-522	28
**p-4**	parallel	3.7	188	6

Table 3 and Fig 9 show that the values of the transient charge signals on the surface, as well as the loading stress, reach peak values during the main rupture of the coal samples. As the limiting stress (*F*) is different, the maximum value of the surface charge signals (*ΔQ*) is also different, indicating that the amount of free charge generated in the surrounding areas of the copper electrode varies in different coal samples placed under uniaxial stress. This result is related to the coal properties and crack propagation near the electrode during the deformation process.

By comparing the results of samples having different cracking propagation, it is found that the bedding direction has a significant effect on the limiting stress and the transient charge signals of the samples. The limiting stress of the samples perpendicular to the bedding fluctuates in the range of 4.6 MPa to 10.2 MPa, and the surface charge signals range from 560 pc to 5117 pc when the main rupture occurs. The number of effective signal pulses generated during the whole loading process is only 2 to 4. In contrast, the limiting stress of the samples parallel to the bedding is 2.1 MPa to 5.3 MPa, clearly smaller than that of the samples perpendicular to the bedding, demonstrating that the direction of parallel bedding reduces the uniaxial strength of the raw coal sample. When the samples parallel to the bedding rupture, the range of the surface charge signals is 188 pc to 590 pc, which is much smaller than the charge signals generated in the samples perpendicular to the bedding. The number of effective signal pulses in the loading process is 6 to 32, which is more than that in the other group of coal samples, indicating that there are more local failures in the samples parallel to the bedding under uniaxial stress.

## Discussion

### Transient charge signals during different failure processes of coal

According to the stress-time curves and the transient charge signals-time curves of the coal samples, the loading process of coal samples can be divided into four stages regardless of the different cracking propagation: load, linear elastic, elastoplastic, and limiting failure [33]. The generating process and inducing factors of the surface charge signals in coal show some differences at each stage.

The first load stage is the sample compaction stage. Raw coal samples are composed of mineral particles, other minerals, and cements joined by Van der Waals forces and contain a large number of pores and cracks. In the first stage, the growth rate of the loading stress is relatively low, and the fracture structure in the samples is compacted under stress. There is sliding friction between adjacent mineral particles or between the mineral particles and any impurities present, and a small number of microcracks are generated to produce free charge during the loading process. As the crack propagation speed is high, the free charges do not completely dissipate, resulting in changes of the internal charge signals in the sample. However, due to the small number of cracks generated at the beginning of the loading period and the low electrical conductivity of coal.Therefore, the transient charge signals on the coal surface (*ΔQ*) generated in this stage only weakly fluctuate and are not effectively collected by the electrode.

In the linear elastic and elastoplastic stages, the growth rate of stress increases, and a certain number of micro ruptures form and result in several local failure events in the coal body. During the process of micro rupture occurrence, the chemical bonds between the coal atoms at the crack tips are broken under stress concentration. As the chemical bonds are formed by electronic interaction, a separation charge will be generated and accumulate on both sides of the crack wall when the cracks propagate because the equilibrium state between electrons is broken, which has been indicated in previous studies [34–35]. Due to the large number of micro ruptures being formed at this stage, a certain amount of free charge can be accumulated and transferred to the surface of the sample, resulting in a sudden change in the transient charge signals received in a short period of time. Fig 6 shows that this stage is more obvious in the samples oriented parallel to the bedding because of the influence of bedding direction. When the loading stress increases to the rapidly increasing stage, the surface charge signals are more intense, and their amplitude is larger.

The final stage is the limiting failure and destabilization stage. There is accelerated deformation in the coal body at this stage, leading to failure and main rupture phase of the sample. The original cracks merge with newer cracks and macroscopic failure causes the coal body to rupture and to release elastic energy. In a very short period of time, a large amount of free charge is produced at the crack tips and transmitted to the surface of the coal body, resulting in a sharp change in the amplitude and polarity of the surface charge signals. After the main rupture, the charge signals decay to an equilibrium state at a relatively low rate, and surface charge signals are no longer being generated.

**Fig 10:**
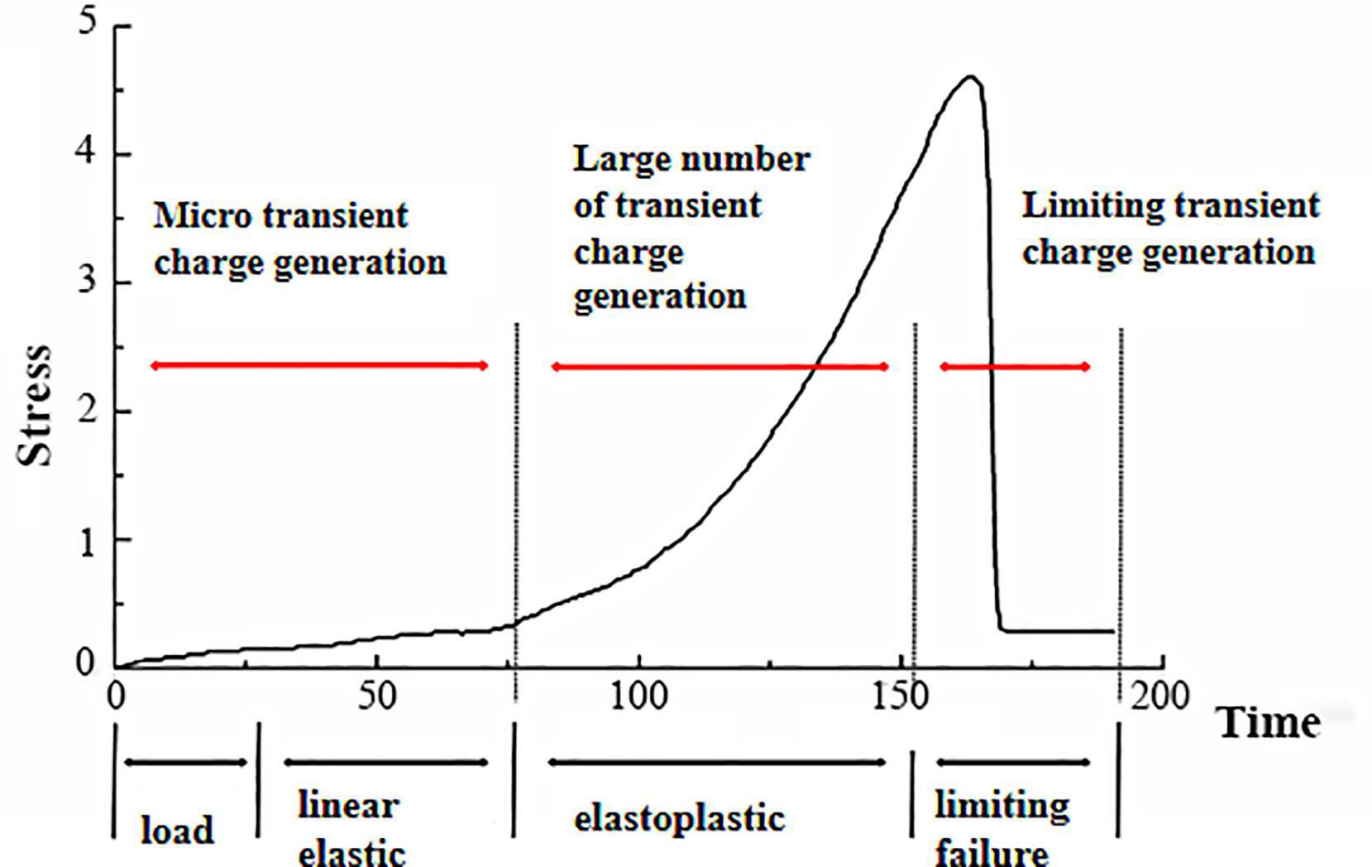
The relationship between transient charge and the failure process of coal.

The relationship between transient charge and failure process of coal is shown in Fig 10.

The above analysis demonstrates that there are some differences in the processes and mechanisms for generating transient charges on the loaded surface of the sample in each stage. The most important reason for the surface transient charge during the damage of the sample is that cracks are generated inside the sample under the influence of stress, and that chemical bonds at the crack tips caused by crack propagation are broken, resulting in charge separation. Internal damage is more obvious with higher sample loading. The more and the faster the crack propagates, the greater is the free charge generated. When a free charge is transferred to the surface of the sample, an instantaneous change in the amount of surface charge occurs. This mechanism of action is most evident when the coal sample is unstable and destroyed, but it should be noted that, before the main rupture of the sample, there is also a transient charge signal, indicating that the charge signal is not completely synchronized with the rupture. Although the friction between the particles at the initial stage of loading is weak, a small amount of transient charge is generated, indicating that friction does play a leading role in this stage and, when the stress concentration increases inside the sample, the free electrons are sufficient. This energy generates escape and a certain concentration of positive and negative charges is formed in the stress concentration region. The experimental results show that the main effect of surface transient charge generation is crack propagation during the deformation and fracture process of coal; other factors also have effects, but their impact is small or even negligible.

### The influence of bedding direction on the transient charge signals

From the experimental results and the parameters shown in Table 3, we observe that the transient charge signals on the coal surface produced in the samples perpendicular to the bedding have larger amplitudes than those of the samples parallel to the bedding but are only generated during the main rupture process of coal body, i.e., the number of effective signal pulses is much lower. The value of limiting stress is also higher compared to the limiting stress of the samples parallel to the bedding, which is due to the different cracking propagation. Due to accumulation modes and later structural changes, the coal body becomes an anisotropic heterogeneous body, especially in the two directions of parallel and vertical bedding distribution, as shown in Fig 3. In this subsection, the influence of bedding direction in coal body on the transient charge signals is discussed.

For the samples perpendicular to the bedding, the bedding direction has little effect on the sample strength when the sample is under uniaxial load. During the initial loading process, there are no obvious cracks or local failures on the surface of the sample, while a large amount of elastic energy accumulates within the sample. As a result, both the surface charge signals and the stress show no significant change during the process. However, when the stress increases to a critical value, the sample body undergoes an abrupt failure and the accumulated elastic energy is released completely in a short time period. As a manifestation of energy release, there is a transient change of positive or negative charge during the sample rupture. Free charges are generated and transmitted to the sample surface, causing the surface charge signals to change with a strong polarity reversal.

For the samples parallel to the bedding, the bedding direction increases the heterogeneity of the coal samples when the bedding direction is parallel to the loading direction, which results in varying internal strength in the coal body. The parallel bedding can be regarded as a weak surface in a coal body, which provides a poor connection between the surrounding coal and rock particles and reduces the compressive strength of the sample. Unlike the abrupt failure in the samples perpendicular to the bedding, there are a number of small intensity local failures which occur in the samples parallel to the bedding during the loading process, leading to some elastic energy release. As a result, the samples parallel to the bedding show splitting failure under uniaxial pressure, and the corresponding free charge signals also show small amplitudes and dense characteristics, especially at the beginning of the loading process. It is difficult for energy accumulation to take place during this deformation process. It is obvious that the peak value of the surface charge signal is much smaller than that of the samples oriented perpendicular to the bedding during the main rupture of the sample, which is affected by the initial local failure. The elastic energy accumulated within the sample has already been released, to a certain extent, during the beginning of the compression process. As a result, the energy released during the main rupture process is less than that of the samples perpendicular to the bedding, and the abruptness of rupture is not as striking.

## Conclusions

We experimentally investigated the effect of bedding direction on the transient charge signals on surfaces of coal samples under uniaxial stress. The main findings from this study can be summarized as follows:

1. Transient charge signals are generated on sample surfaces during the deformation process of coal under uniaxial compression, which is consistent with the corresponding stress change. The different cracking propagation have a significant influence on the characteristics of the surface charge signals and on related parameters due to the reduction of uniaxial strength. The charge signals show much larger amplitudes and fewer effective pulses in the samples perpendicular to the bedding than those of the samples parallel to the bedding.
2. The characteristics of the surface charge signals generated at different stages during the loading process of the samples are different. In the sample compaction stage, the surface charge signals are not obvious and fluctuate only weakly. In the elastoplastic deformation stage, a certain amount of local failure occurs in the coal specimens, leading to more surface charge signals exhibiting larger amplitudes. In the destabilization stage, the level of collected surface charge signals rises sharply in a very short period of time, indicating that free charges are generated and are accumulating on the coal surface.
3. The main reason for the change in the surface charge signals is that crack propagation in coal leads to charge separation between the electrons at the crack tips and that free charges are transmitted and accumulate on the coal surface. Both in the elastoplastic deformation stage and in the destabilization stage, a large number of microcracks develop, which provide more effective free charges.
4. The amount of free charges accumulating on the sample surface may change abruptly over a very short time period, and alternation of positive and negative charges takes place along with the changing process. This result indicates that the polarity of the free charges caused by the crack propagation during the rupture will alternate with time and impact the surface charge accumulation, the mechanism of which may need further study.
